# Biological importance of human amniotic membrane in tissue engineering and regenerative medicine

**DOI:** 10.1016/j.mtbio.2023.100790

**Published:** 2023-09-01

**Authors:** Zeming Hu, Yang Luo, Renhao Ni, Yiwei Hu, Fang Yang, Tianyu Du, Yabin Zhu

**Affiliations:** Health Science Center, Ningbo University, Ningbo, 315211, China

**Keywords:** Human amniotic membrane, Properties, Modifications, Tissue engineering, Regenerative medicine

## Abstract

The human amniotic membrane (hAM) is the innermost layer of the placenta. Its distinctive structure and the biological and physical characteristics make it a highly biocompatible material in a variety of regenerative medicine applications. It also acts as a supply of bioactive factors and cells, which indicate the advantages over other tissues. In this review, we firstly discussed the biological properties of hAM-derived cells *in vivo or in vitro*, along with their stemness of markers, pointing out a promising source of stem cells for regenerative medicine. Then, we systematically summarized current knowledge on the collection, preparation, preservation, and decellularization of hAM, as well as their characteristics helping to improve the understanding of applications in tissue engineering. Finally, we highlighted the recent advances in which hAM has undergone additional modifications to achieve an adequate perspective of regenerative medicine applications. More investigations are required in utilizing appropriate modifications to enhance the therapeutic effectiveness of hAM in the future.

## Graphical abstract

The schematic diagram highlights the biological importance of hAM or hAM-derived stem cells, along with their additional modifications, contributing to an outstanding perspective in clinical applications.

**Figure:**
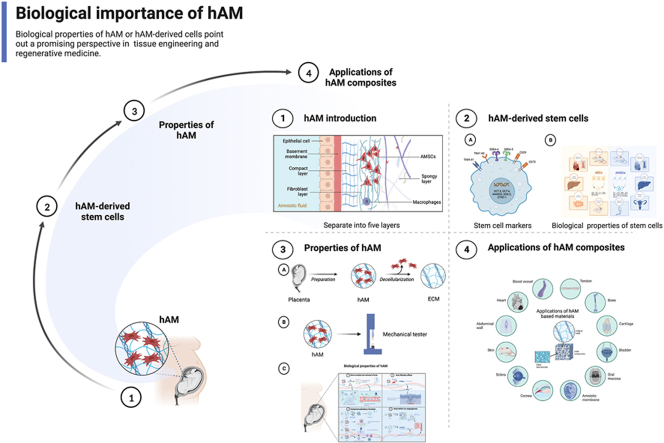

## 1 Introduction

**Fig. 1:**
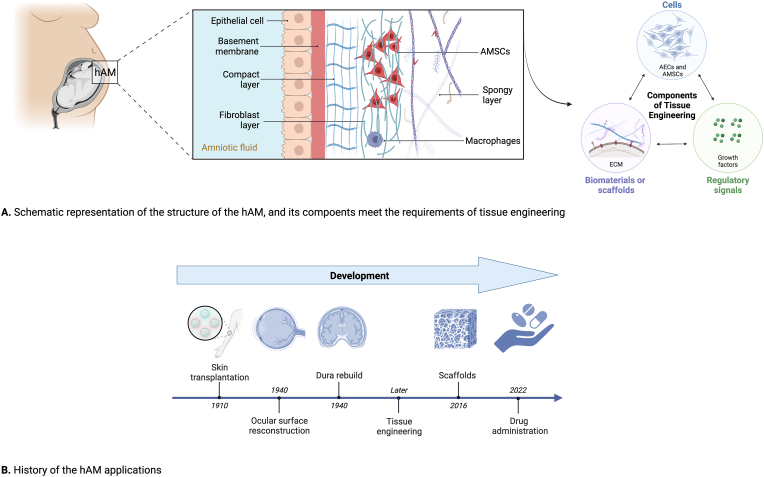
**Human amniotic membrane.** (A) Schematic illustration of hAM anatomy: an epithelial layer, the basement membrane, a compact layer, a fibroblast layer, and a spongy outer layer. The components of hAM fulfill the requirements of tissue engineering, which contains cells, growth factors, and scaffolds. (B) A timeline of hAM for clinical applications over the past century.

The human amniotic membrane (hAM) is the deepest layer of the placenta and is characterized by being a rough, thin (∼0.02–0.5 mm), and semi-transparent membrane without blood vessels, lymphatics, and nerves [1,2]. Like the placenta, umbilical cord, and amniotic fluid, hAM is readily available and regarded as a significant source of stem and progenitor cells. Two main cell types can be found in the hAM, amniotic mesenchymal stromal cells (AMSCs) and amniotic epithelial cells (AECs) [3]. Specifically, AECs is towards the fetus and in direct contact with the amniotic fluid. Meanwhile, the AMSC population is inserted in the extracellular matrix (ECM) of the hAM [4]. The hAM-derived stem cells have multiple characteristics, including immunomodulation, inflammation inhibition, fibrosis and scarring reduction, angiogenesis facilitation, and oxidative stress remission [5,6]. Additionally, hAM can be divided into the following five layers, as Fig. 1A shown; a single epithelial layer toward the fetus, the basement membrane, a compact layer, a fibroblast layer, and a spongy outer layer [7]. These tissues are usually applied as a biological matrix as they fulfill the three main components of the tissue engineering concept: cells, scaffolds, and growth factors.

The epithelial layer, enclosed by amniotic fluid, comprises a single columnar or cuboidal AECs generated from ectoderm [8]. The AECs adhere to the basement membrane, separating them from the compact layer connected with the stromal side [9]. The AECs can generate several growth factors, including epidermal growth factor (EGF), basic fibroblast growth factor (bFGF), vascular endothelial growth factor (VEGF), transforming growth factor (TGF-α/β1-3), and platelet-derived growth factor (PDGF) [10]. The intermediate layer is a thick basement membrane that divides the epithelium and stromal layers, which is morphologically similar to human skin due to their comparable composition like collagen I/II/III/IV, laminin, and fibronectin [2]. It is one of the thickest membranes in the human body, offering tensile strength, structural integrity and mechanical characteristics. It indicates that these characteristics pertain to the stromal layer of the membrane [8,11]. Moreover, the basement membrane contains some proteins, including collagen I/II/III/IV, laminin-1/5, and fibronectin [12], besides comprising hyaluronic acid and critical ECM proteins, forming and functioning as an ECM [13].

The stromal layer (the mesenchymal layer) is the outer hAM layer and comprises connective tissues. It is the thickest layer in hAM, including three sub-layers from the inside to the outside: an acellular compact layer composed of reticular fibers, including collagen I/III secreted by AMSCs, a fibroblast layer composed of a loose reticular network containing dispersed fibroblast-like mesenchymal stromal cells and sporadic macrophages, and a spongy layer that is in touch with the chorion membrane and has a complicated network of adequate fibrils wrapped in mucus [14,15]. This stromal layer is transparent due to its lack of vascularization and innervation [9].

The ECM of hAM comprises various collagens, laminin, proteoglycans, glycoproteins, elastin, and fibronectin, besides various significant growth factors and cytokines [16]. It has been used as a natural biomaterial and as a possible cell source for various therapeutic purposes [17,18]. For example, Sabklla N et al. [19] and Stern M et al. [20] advocated its application in skin transplantation in early 1910. In 1940, De Rötth et al. [21] advocated its utilization to replace injured conjunctiva instead of the mouth mucous membrane due to its thin, smooth, and translucent structure that matches the original conjunctiva tissue. Chao et al. [22] successfully rebuilt the dura in an experimental severe head injury using hAM as the matrix. Since then, hAM has been gained momentum as a new biomaterial in many other fields owing to its significant capabilities as a scaffold for blood vessels or other tissue regeneration [23]. Moreover, it can be improved by adding growth factors and cytokines in distinct applications in regenerative medicine, three-dimensional (3D) scaffolds for tissue regeneration, and drug administration [24]. Fig. 1B presents a timeline of the hAM development for research throughout the past century.

Therefore, many beneficial features of hAM, including availability, low cost, and isolation simplicity, make it a suitable biomaterial in tissue engineering researches, particularly in the engineered skin and other soft tissues [25,26]. Moreover, hAM has excellent biological properties, including antibacterial, anti-scarring, immunomodulatory, site-dependent angiogenic functions, and low immunogenicity [27,28]. Thereby, it has been extensively exploited in tissue engineering and regenerative medicine. This review aims to summarize the biological and mechanical properties of hAM and the characteristics of hAM-derived stem cells. In addition, the modifications of hAM for the applications in regenerative medicine were discussed.

## 2 hAM-derived stem cells

Stem cells are essential in regenerative medicine due to their self-renewal, proliferation, and differentiation abilities. Many investigations have shown that the hAM includes cells having stem cell properties. The primary stem cell types are AECs which rest on a basement membrane, and AMSCs, which reside in the fibroblast layer of the membrane [29,30]. Both cell types have embryological origin characteristics and generate before the three germ layers formation in the pre-gastrulation stages [31]. The AECs are formed from the pluripotent epiblast on the eighth day of conception, while the AMSCs are generated from the extraembryonic mesoderm of the primitive streak [32].

Additionally, hAM-derived stem cells are simple to isolate, where AECs present a cobblestone-like morphology, and AMSCs display a fibroblast-like morphology *in vitro* [[33], [34], [35]]. Compared with other stem cell sources, hAM-derived stem cells have several advantages such as easy availability, non-tumorigenicity, low immunogenicity, high histocompatibility, effective paracrine function and the minimal ethical concerns. These advantages enable the hAM-derived stem cells to be highly appealed to people, and thus achieve the opportunity to be used as cells in cell therapy and regenerative medicine in the clinic [6,36,37].

When exposed to appropriate culture conditions, hAM-derived stem cells can differentiate into cell lineages of all three primary germ layers [38]. Both AMSCs and AECs display the conventional embryonic stem cell markers, including SSEA-3/4, TRA1-60/81, particularly the pluripotent markers of SOX-2, OCT-4, and NANOG, revealing their potential for regenerative medicine [34,39]. Most hAM-generated cells express embryonic stem cell markers like TRA1-60/81 and SSEA-3/4 in the early second trimester [40]. Similarly, most AECs and AMSCs display certain embryonic stem cells associated pluripotent markers in the early stage, including SSEA-3/4, OCT-4, NANOG, and TRA1-60/80 [41].

Depending on the profiling of different stem cell markers, hAM-derived stem cells represent a heterogeneous cell population [42]. For instance, the transcription factor NANOG, which is considered the most stringent marker for stem cells, is expressed in only 1–3% of AECs, while ∼50% of AECs are SSEA-4-positive at gestation [41]. Only 4% of hAM-derived cells co-express the stem cell markers SSEA-4 and TRA1-60/81 [43]. Stem marker-positive AECs are distributed indiscriminately over the whole hAM rather than residing in clusters and seem surrounded by marker-negative cells [44,45]. The pluripotent marker expressions and proliferative capacity of AECs from distinct hAM regions are very different [46]. This indicates that the AECs encompass the whole epithelial layer at different gestational stages with distinct gene expressions and stemness degrees. Additionally, most primary hAM-derived stem cells may be oriented toward a particular differentiation *in vitro*, despite the absence of stem cell markers [47].

Besides, the typical mesenchymal stem cells (MSCs) markers CD44/73/90/105 are expressed within AMSCs and AECs, whereas CD34/45/80/86 and HLA-CR are not expressed [31]. MSC markers in AMSCs suggest that the cells have immune privilege and immunomodulatory properties, desirable therapeutic advantages in the medical field [48]. This indicates that the immune protection of AMSCs may contribute to immune system suppression or modulation. Table 1 lists the stem cell markers of AECs and AMSCs.

**Table 1:** Stem cell markers of AECs and AMSCs generated from the hAM.

Cell type	MSCs markers	Cell surface markers	Transcription factors	Reference
AECs	CD29/73/105	SSEA-4	OCT-4 and NANOG	[35]
	CD9/24	SSEA-3/4 and TRA1-60/81	OCT-4 and NANOG	[42]
	CD29/90/166	TRA1-60	OCT-4, NANOG, and SOX-2	[56]
	CD29/44/73/90/105	SSEA-4	OCT-4 and SOX-2	[57]
	CD29/44/90	SSEA-4	OCT-4 and SOX-2	[58]
	CD29/73	SSEA-4	OCT-3/4 and SOX-2	[59]
AMSCs	CD29/73/90/105	SSEA-4	OCT-4 and NANOG	[34]
	CD29/44/73/90/105	SSEA-4	OCT-4 and SOX2	[57]
	CD44/29/73/90/105/271	SSEA-4 and TRA1-60	OCT-3/4, SOX-2, and NANOG	[59]
	CD29/44/73/90/105	SSEA-4	OCT-4	[60]
	CD29/44/73/90/105166	SSEA-4	STRO-1	[61]
	CD29/105	SSEA-4 and REX-1	OCT-3/4, SOX-2, and NANOG	[62]
	CD44/73/90/105	SSEA-3/4	OCT-3/4, SOX-2, and NANOG	[63]

The lack of immunogenicity is widely recognized as a prominent characteristic of hAM-derived cells, resulting in their state of immunological tolerance [49]. Regarding innate immune suppression, AECs and their conditioned medium (CM) substantially reduce T-cell proliferation with or without direct contact [50]. Moreover, AECs and AMSCs have indicated their ability to inhibit T-cell proliferation in a dose-dependent manner, which was observed after T-cell exposure to alloantigen in the presence of hAM-derived stem cells, either after CD3/28 stimulation or in typical mixed lymphocyte response models [51,52]. The tolerogenic HLA-G expression is a crucial factor in the immunomodulatory abilities of hAM-derived stem cells. HLA-G interacts with the appropriate immune cell receptors (ILT2/4 and KIR2DL4) to exert immunomodulatory effects [53]. Furthermore, the immunological checkpoint proteins like programmed death ligands 1 and 2 (PD-L1/2), are associated with immune tolerance induction by hAM-generated cells [54]. These molecules are present on AMSCs and syncytiotrophoblasts in the placenta. However, they can be synthesized by AECs without being exposed to IFN-γ [55].

### 2.1 hAM-derived AECs

AECs' migration to damaged tissues to replace the injured cell is essential to ameliorate acute or chronic wounds. Nevertheless, there are several challenges to stem cell transplantation, including low survival and poor repair capacity. Recently, AECs may offer a favorable microenvironment for cell survival and trigger endogenous tissue regeneration by secreting bioactive cytokines, growth factors, and exosomes [64]. AECs could synthesize and release brain-derived neurotrophic factor (BDNF), ciliary neurotrophic factor (CNTF), and forskolin. These factors are involved in embryonic neural development at early stages and have a neuroprotective function [65].

Additionally, literature [66] revealed that AECs increased the lifespan of dopamine (DA) neurons by generating biologically active neurotrophins, including BDNF and neurotrophin-3 (NT-3), preventing DA neurons loss in rats with Parkinson's disease. Regarding corneal injury, AECs-CM injection enhanced wound healing by lowering inflammatory cell infiltration and neovascularization [67]. The AECs-CM injection also improved the ovarian microenvironment, defended the ovaries against the harm caused by chemotherapy, and promoted ovarian angiogenesis [68]. Furthermore, AECs effectively reduced chemotherapy-induced apoptosis and activated the TGF-β signaling pathway in primary granulosa-lutein cells in a paracrine manner [69]. The AECs-CM injection also promoted angiogenesis in mice with premature ovarian failure/insufficiency in the injured ovaries.

On the other hand, AECs reduced collagen synthesis in human hepatic stellate cells with paracrine effect, and some soluble factors like MMP-2/9 were secreted by AECs, enhancing ECM remodeling, reducing liver fibrosis, and altering macrophage polarization, particularly in liver fibrosis models [70]. Administrating AECs and AECs-CM decreased hepatic inflammation and fibrosis significantly in a diet-induced non-alcoholic steatohepatitis model, indicating the anti-fibrotic effects of AECs [71]. Under hypoxic conditions, AECs secrete higher human-origin proangiogenic cytokine levels, including angiogenin (ANG), EGF, interleukin (IL)-6, and monocyte chemoattractant protein (MCP)-1, contributing to myocardial tissue regeneration in a myocardial infarction rat model [72].

Extracellular vesicles, specifically exosomes, have received much attention as they are essential components of AECs paracrine effects. Exosomes are nano-sized microvesicles containing multiple bioactive molecules, including proteins, nucleic acids, lipids, and organelles. These vesicles are involved in intercellular communication and regulate many intracellular biological activities [73]. Internalization of AECs-derived exosomes enhanced fibroblast migration and proliferation, partly abolished ECM deposition, and improved cutaneous wound healing within *in vivo* experiments [74]. In idiopathic pulmonary fibrosis, AECs-derived exosomes polarized and enhanced macrophage phagocytosis, reduced neutrophil myeloperoxidase activity, and directly suppressed T-cell proliferation, eventually improving lung fibrosis [75]. Moreover, AECs-derived exosomes increased the number of follicles and enhanced ovarian function in a chemotherapy-induced premature ovarian failure mice model. Another *in vitro* study showed that AECs-derived exosomes prevented chemotherapy-induced apoptosis in granulosa cells by transmitting miR-1246 [76]. In brief, there has been a systematic review of the biological functions of AECs secreted exosomes or extracellular vesicles in regenerative medicine [77].

### 2.2 hAM-derived AMSCs

Currently, AMSCs originate from the hAM and are regarded as highly intriguing stem cells in regenerative medicine. AMSCs contribute to treating skin wounds due to their multilineage differentiation, immunosuppressive, and anti-inflammatory capabilities [78]. AMSCs and AMSCs-CM prevented heat stress-induced apoptosis in human keratinocytes and dermal fibroblasts and enhanced their proliferation with the paracrine effect. Moreover, AMSCs could secrete many bioactive cytokines, including PAI-1, C-GSF, TIMP-1, and uPAR, which may activate the PI3K/AKT signaling pathway [34]. Additionally, AMSCs-CM significantly accelerated wound closure by secreting IGF-1, EGF, and IL-8 in a diabetic mice model [79].

The AMSCs exhibit unique characteristics that render them valuable in tissue regeneration applications. For example, AMSCs combined with PPCNg (thermoresponsive biomaterial composed of Poly (polyethylene glycol citrate-co-N-isopropyl acrylamide) (PPCN) mixed with gelatin) intrauterine transplantation could promote damaged endometrium regeneration, restoring reproductive function in an intrauterine adhesions rat model [80]. PPCNg has been used as a scaffold for stem cell transplantation. Additionally, AMSCs injection significantly restored ovarian function in an age-related diminished ovarian reserve (AR-DOR) mice model while dramatically suppressing granulosa and stromal cell apoptosis in the ovaries [81]. In a carbon tetrachloride-induced liver fibrosis mice model, IGFBP-3 and DKK1/3 generated from AMSCs prevented the activation of hepatic stellate cells by suppressing the Wnt/β-catenin signaling pathway, decreasing liver fibrosis in mice [82]. These findings suggested that AMSCs or AMSC-CM could provide an alternative therapeutic strategy for treating hepatic fibrosis. Moreover, AMSCs transplantation might reverse Bmi-1 deficiency-induced mandibular osteoporosis by promoting osteoblastic bone formation and preventing bone resorption [83].

The proangiogenic potential of AMSCs has been investigated in many publications due to exhibiting high levels of the proangiogenic genes VEGF-A, angiopoietin-1, HGF, FGF-2, and the antiapoptotic protein AKT-1. Administrating AMSCs increased blood flow and capillary density in hindlimb ischemia of mice, demonstrating that AMSCs significantly enhanced neovascularization [84]. Similarly, AMSCs-CM significantly recovered the blood flow in a murine hindlimb ischemia model [85]. Intramyocardial injection of AMSCs might restore cardiac function and suppress inflammation at the damage site by regulating the inflammatory MAPK/NF-κB pathway in a myocardial injury rat model [86]. Furthermore, superparamagnetic iron oxide nanoparticles-labeled AMSCs dramatically enhanced cardiac function and decreased fibrosis and tissue injury in a magnetic field by inhibiting inflammation in an NF-κB/MAPK-dependent manner [87].

**Fig. 2:**
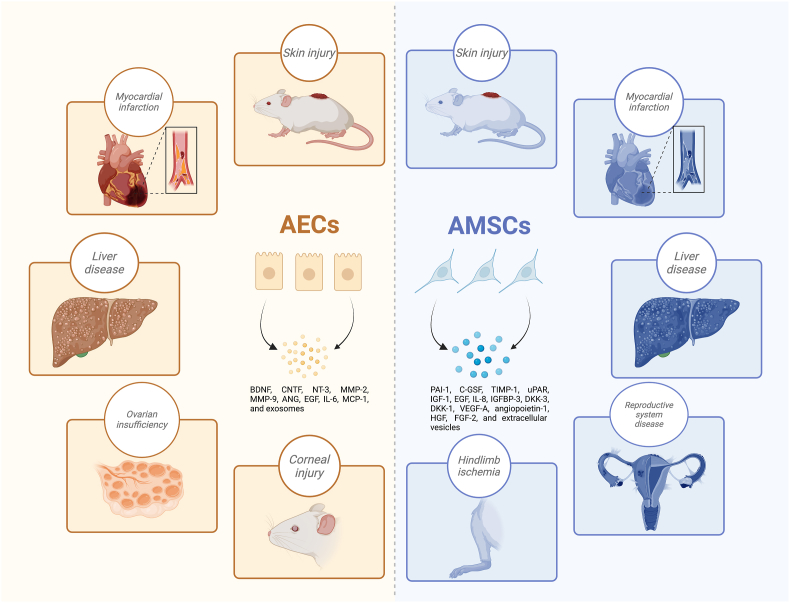
**The biological effects of hAM-derived AECs and AMSCs in various disease models.** AECs and AMSCs display similar therapeutic efficiency in animal models of reproductive system disease, liver disease, myocardial infarction, corneal injury, hindlimb ischemia, and skin injury.

Moreover, *in vivo* and *in vitro* results revealed that the derivatives and protein extracts of the AMSCs present potential anti-cancer effects. For instance, DKK-1/3 and IGFBP-3 generated from AMSCs suppressed hepatoma cell proliferation and induced apoptosis by inhibiting specific signaling pathways [33]. The anti-tumor effects of AMSCs on prostate cancer cells were also identified by activating apoptosis, inhibiting the epithelial-mesenchymal transition process, and down-regulating EGFR using 2D and 3D cell culture methods [88]. The immunomodulatory characteristics of AMSCs secreted exosomes or extracellular vesicles enable them to promote tissue regeneration and produce therapeutic benefits in preclinical disease models. AMSCs performed immunoregulatory functions via the paracrine effect activated by factors in their extracellular vesicles [89]. This suggests that extracellular vesicles generated from AMSCs inhibited peripheral blood mononuclear cell proliferation and T-cell polarization toward inflammatory Th subtypes, besides stimulating regulatory T-cell activation. The biological characteristics of AECs and AMSCs are summarized in Table 2, and the applications in tissue engineering are diagrammatically displayed in Fig. 2.

**Table 2:** hAM-derived stem cells display biological functions in various disease treatments.

Stem cells	Secreted factors/cytokines	Biological functions	Experimental cells or animals	Involved diseases	Reference
AECs	BDNF, CNTF and forskolin	Neuroprotective effect	Rat retinal ganglion cells	N/A	[65]
AECs	BDNF and NT-3	Enhance DA neurons survival	Rats	Parkinson's disease	[66]
AECs-CM	N/A	Anti-inflammatory effect	Rabbits	Corneal injury	[67]
AECs-CM	VEGF	Promote angiogenesis	Mice	Chemotherapy-induced primary ovarian insufficiency	[68]
AECs and AECs-CM	TGF-β	Anti-apoptosis and promote angiogenesis	Primary granulosa-lutein cells, mice	Premature ovarian failure/insufficiency	[69]
AECs and AECs-CM	MMP-2/9	Enhance ECM remodeling, anti-fibrosis, and alter macrophage polarization	Human hepatic stellate cells	Liver fibrosis	[70]
AECs and AECs-CM	MMP-2/9	Anti-inflammation and anti-fibrosis	Mice	Non-alcoholic steatohepatitis	[71]
AECs	ANG, EGF, IL-6, and MCP-1	Promote angiogenesis	Rats	Myocardial infarction	[72]
AECs-derived exosomes	MMP-1	Enhance fibroblast migration and proliferation, and downregulate collagen expression	Fibroblasts and rats	Skin injury	[74]
AECs-derived exosomes	miRNA	Enhance macrophage phagocytosis, reduce neutrophil myeloperoxidases activity, and suppress T cell proliferation	Mice	Idiopathic pulmonary fibrosis	[75]
AECs-derived exosomes	miR-1246	Anti-apoptosis	Granulosa cells and Mice	Chemotherapy-induced premature ovarian failure	[76]
AMSCs and AMSCs-CM	PAI-1, C-GSF, TIMP-1, and uPAR	Anti-apoptosis and pro-proliferation	Keratinocytes, dermal fibroblasts, and Mice	Skin wound	[34]
AMSCs	IGF-1, EGF, and IL-8	Promote angiogenesis	Mice	Diabetic skin wound	[79]
AMSCs	VEGF and Ki-67	Promote angiogenesis and pro-proliferation	Rats	Intrauterine adhesions	[80]
AMSCs	N/A	Anti-apoptosis	Granulosa cells and Mice	Age-related diminished ovarian reserve	[81]
AMSCs and AMSCs-CM	IGFBP-3 and DKK-1/3	Inhibit the collagen deposition and anti-fibrosis	Hepatic stellate cells and Mice	Carbon tetrachloride-induced liver fibrosis	[82]
AMSCs	N/A	Stimulate the osteoblastic bone formation	Mice	Mandibular osteoporosis	[83]
AMSCs	VEGF-A, angiopoietin-1, and FGF-2	Promote angiogenesis	Mice	Hindlimb ischemia	[84]
AMSCs-CM	N/A	Promote angiogenesis	Mice	Hindlimb ischemia	[85]
AMSCs	N/A	Anti-inflammation	Rats	Myocardial injury	[86]
AMSCs	N/A	Anti-fibrosis	Rats	Isoproterenol-induced myocardial injury	[87]
AMSCs	DKK-1/3 and IGFBP-3	Anti-tumor effect	Hepatoma cells	Liver cancer	[33]
AMSCs	N/A	Anti-tumor effect	Prostate cancer cells	Prostate cancer	[88]
AMSCs-derived extracellular vesicles	N/A	Immunomodulatory effect	Immune cells	N/A	[89]

## 3 Processing and properties of hAM-derived materials

The interdisciplinary fields of tissue engineering include process engineering, material science, biology, chemistry, physics, and medicine. It focuses on fabricating tissue analogs to support cellular responses in successfully regenerating lost or injured tissues and organs [28]. Tissue engineering consists of cells, scaffolds, and growth factors or biomolecules that stimulate tissue regeneration. The primary concept underlying tissue engineering is creating a new effective scaffold or scaffold-based tissue structure in tissue-engineered constructions or products. Tissue-engineered constructions are created by colonizing a scaffold with cells that can proliferate, differentiate, and duplicate the functions of cells or/and tissues [90]. The scaffold is a cell adhesion and infiltration platform to produce functional tissue. Consequently, the cells need a biocompatible scaffold made of natural, synthetic, or a combination of both materials that resemble the ECM [91]. The optimal scaffolds should be biodegradable, meaning they should degrade non-toxic when implanted into the impaired tissue microenvironment and be replaced by native components after tissue repair is finished [92].

The hAM is a biomaterial with multiple appealing properties. There are no ethical issues since it is considered biological waste after delivery. The availability, affordability, and simplicity of isolation make hAM a suitable biomaterial for tissue engineering [93]. Additionally, the hAM can be extensively employed in tissue engineering due to its favorable biological characteristics. The properties of hAM with different forms are summarized in Table 3.

**Table 3:** Properties of hAM with different forms.

Characteristics	Specific features	Forms	Mechanism	Study mode	Reference
Mechanical properties	Elasticity	Intact hAM	Young's modulus (Full term hAM: 2.29 MPa; preterm hAM: 3.6 MPa)	*In vitro*	[13]
	Stiffness	Intact hAM	Elastic modulus: 4.048 ± 1.702 MPa	*In vitro*	[110]
	Tensile strength	Dehydrated hAM	5.475 ± 0.135 MPa	*In vitro*	[111]
	Stiffness	Decellularized hAM	Elastic modulus higher than native hAM	*In vitro*	[28]
Biological properties	Antimicrobial effect	Intact hAM	Expression of elafin, HBD-2/3, and cathelicidin LL-37	*In vivo*	[112]
	Antimicrobial effect	Cryopreserved hAM	Expression of HBD-2/3	*In vivo*	[113]
	Antimicrobial effect	hAM extract	Secretion of antimicrobial proteins and peptides	*In vitro and in vitro*	[114]
	Antimicrobial effect	hAM homogenate	Expression of β-defenses	*In vitro*	[115]
	Antiviral effect	Intact hAM	Expression of IL-1RA	Clinical cases	[117]
	Antiviral effect	Intact hAM	Inhibition of inflammatory response	Clinical cases	[118]
	Anti-fibrotic effect	hAM extract	Reversion to the fibroblast phenotype	*In vitro*	[122]
	Anti-scarring effect	Intact hAM	Antagonizing the effect of TGF-β	*In vitro*	[123]
	Anti-inflammatory effect	Decellularized hAM	Suppression of IL-1α/1β	*In vitro*	[125]
	Anti-inflammatory effect	Intact hAM	Trapping inflammatory cells	Clinical cases	[126]
	Low immunogenicity	Intact hAM	Expression of HLA-G and Fas ligand	*In vivo*	[127]
	Immunomodulatory effect	Intact hAM	Proliferation and induction of immune cells	*In vitro*	[128]
	Anti-angiogenesis effect	Flesh hAM	Secretion of collagen IV, laminin, and integrins 4/6	*In vivo*	[130]
	Anti-angiogenesis effect	Intact hAM	Secretion of endostatin, thrombospondin-1, and TIMP-1/2/3/4	Clinical trial	[131,132]
	Angiogenesis effect	Intact hAM	Secretion of fibronectin	*In vitro*	[133]
	Angiogenesis effect	Decellularized hAM	Enhancing PECAM-1 and VE-cadherin expression	*In vitro*	[134]
	Support cell growth and adhesion	Decellularized hAM	Expression fibronectin, laminin, collagens, and proteoglycans	*In vitro*	[135]

### 3.1 Collection, preparation, preservation, and decellularization

Generally, hAM is acquired from the human placenta and taken by medical faculty following a cesarean with informed consent from expectant mothers. hAM could also be obtained from a natural delivery. The source of hAM collection, whether after vaginal delivery or via cesarean section, influence its physiology, integrity, availability, and growth factor content and has a contamination risk. The hAM donors should be screened to minimize infectious disease transmission, particularly after the COVID-19 pandemic owing to the concerns about vertical transmission [94]. After collection, the placenta is packaged in a sterile container and transferred to the laboratory for processing. The transport duration should be instant, within 24 h at 4 °C or 2 h at room temperature [28]. It should be immersed in sterile saline solution or phosphate-buffered saline to prevent the hAM from dehydration [95].

The preservation of hAM has been developed over the last century benefiting from technical advances, preparation methods, and sterilizing procedures. In 1940, Chao et al. [22] advocated drying hAM before usage to reduce discomfort caused by fresh hAM. Dry hAM marked a turning point when used in tissue reconstruction since it proved safe and addressed the shortcoming of fresh hAM. Later, Kim et al. [96] suggested cryopreservation of the hAM by storing it at −80 °C using a storage medium composed of glycerol and Dulbecco's Modified Eagle Medium (DMEM). However, cryopreservation altered the composition and distribution of ECM, leading to poor cell viability in the hAM. Moreover, cryopreservation reduces the number of growth factors or cytokines produced by hAM, endangering its structural and mechanical features [2]. In 2004, Nakamura et al. [97] presented a novel preservation method, freeze-dried hAM as a substrate for ocular surface reconstruction, to confirm the feasibility. Both cryopreservation and lyophilization have drawbacks since they could influence hAM characteristics. Current efforts to preserve the hAM in suspension have shown the promise in reducing the invasiveness, comparing with the grafting process of suturing the hAM, particularly for corneal ulcers [24]. This suspension has been commercialized as a cryopreserved amniotic membrane extract (AME) or as AME-eye drops. The cryopreserved AME has been shown to alleviate the clinical manifestations of dry eye disease [98].

Collectively, the hAM is currently utilized in various forms, including fresh, air-dried, freeze-dried, cryopreserved, and suspension. Due to the rejection response of hAM allografts after transplantation, intact hAM immunogenicity remains a serious problem [15]. The decellularization of hAM before transplantation is considered a highly effective method.

Decellularization involves removing the cells and cell debris from the hAM and leaving the extracellular structural proteins unchanged to avoid immunogenic reactions and reduce the risk of infection [99]. The acellular hAM has been shown to retain ECM components, including collagen I/III/IV/V/VII, laminin, fibronectin, elastin, hyaluronic acid, and several bioactive factors. The decellularized hAM produces multiple growth factors such as NGF, EGF, HGF, KGF, and bFGF accumulating in the stromal layer of the membrane [100]. Each of these growth factors can significantly accelerate tissue regeneration. Besides providing a supporting scaffold with minimal immunogenicity, the decellularized hAM stimulates cell growth, adhesion, and proliferation [101]. Consequently, an acellularized hAM may represent a more suitable and biocompatible alternative for regenerative medicine applications than intact hAM.

Successful decellularization of the hAM while minimizing any adverse impact on the ECM components is vital to prevent immunogenic response after transplantation. The effectiveness of the decellularized procedure is assessed using optical microscopy, immunohistochemistry analyses, fluorescent staining, or DNA quantitative analysis [28]. Generally, most manufacturing methods of decellularized hAM involve two steps under sterile conditions. Briefly, hAM is treated with an enzymatic or chemical reagent, followed by mechanical scraping under light microscopy to remove loosened cells. There are different reagents for hAM decellularization, including sodium dodecyl sulfate, urea, methanol, chloroform, EDTA, and trypsin [102]. However, mechanical scraping may severely damage the integrity of the basement membrane and stroma layer. Therefore, recent research has proposed other strategies that obviate further mechanical scraping [9]. Additionally, decellularization removes the stem cell components from the hAM. Residual non-nuclear cellular components may be found in the ECM; however, their presence is not expected to prevent ECM therapeutic usage.

### 3.2 Mechanical properties

The mechanical properties of hAM, including elasticity, stiffness, and tensile strength, are related to its ECM components. Elastic deformation is determined by the presence of elastin fibers, laminin, hyaluronic acid, and glycosaminoglycan in the ECM. Meanwhile, the collagen fibrils composition determines tensile strength [103]. The hAM displays viscoelastic characteristics and time-dependent mechanical properties depending on its term stage. For instance, as compared to full-term hAM (36–40 weeks), the preterm (26–36 weeks) hAM was shown to have better mechanical integrity [104]. A study further confirmed that Young's modulus of the full-term and preterm hAM were 2.29 and 3.36 Mpa, respectively [13]. Young's modulus is characterized by the elasticity of hAM, defined as a biomaterial's capacity to endure a force that causes distortion and regains its initial form and size when the force is withdrawn.

In addition, it has been proven that the stiffness decreases with the increase of hAM thickness. According to its anatomical structure, hAM thickness varies from 0.02 to 0.5 mm and can be divided into different subregions, known as the placental, reflected and umbilical amnion which differ distinctively in morphology [18,105,106]. A specific area within the reflected amnion is found at the lower uterine pole and cervix, known as the cervical amnion [107,108]. The thickest subregion is adjacent to the placental disk (placental amnion), while the distal subregion (cervical amnion) is thinner and more transparent [109]. Consistently, the placental amnion has a lower elastic modulus (stiffness of biomaterial) than the cervical amnion, which could be attributed to collagen fiber arrangement [110]. Besides differences in stiffness between hAM subregions, differences in tensile strength have also been studied. The placental amnion was significantly more substantial and stretchable than the reflected amnion by an average of 82% ± 45% and 19% ± 6%, respectively [111]. The differences in the elastic modulus of hAM are caused by the various hAM forms utilized. Decellularization of hAM displays a higher elastic modulus than the native one, a possible explanation may be attributed to the denudation process dehydrating the membrane and then decreasing its thickness [28].

### 3.3 Biological properties

As mentioned above, the hAM has shown promising biological properties that enhance various applications in tissue engineering and regenerative medicine. Regarding the antibacterial capabilities of hAM, a study demonstrated that hAM secreted more antimicrobial peptides, including elafin, HBD-2/3, and cathelicidin LL-37, when exposed to IL-1β [112]. This confirms the antibacterial activity, which inflammatory signal inducers can augment. Recently, it has shown that a cryopreserved hAM may efficiently promote wound closure and prevent wound-related infections [113]. Another study declared that the hAM extract suppressed *S. pneumoniae* proliferation in the planktonic and biofilm states by secreting antimicrobial proteins and peptides [114]. In the same direction, Taja et al. [115] showed that the hAM homogenate exhibited an antimicrobial effect against 7 of 11 tested multidrug-resistant pathogens, with methicillin-resistant *S. aureus* having the most significant impact.

There are currently a few studies on the antimicrobial and antiviral effects of hAM. Notably, hAM can exert antiviral properties by restricting either virus colonization and reproduction or severe consequences caused by an abnormal host response to infection, such as sepsis [116]. A study including 18 patients with herpetic keratitis-related corneal ulcers or non-healing epithelial abnormalities was treated with hAM transplantation. The findings indicated that in the individuals with herpes simplex virus type 1 (HSV-1) keratitis, hAM transplantation enhanced rapid epithelial healing and inhibited stromal inflammation and ulceration. The advantages of hAM in those individuals may be attributed to hAM secreting IL-1RA, which is IL-1α′s natural antagonist [117]. In another study, transplantation of multilayer hAM was employed to treat 15 patients' eyes with herpes necrotizing stromal keratitis that had not healed using topical and systemic aciclovir therapy. After 14 days post-transplantation, all patients presented with an intact epithelial corneal surface and a clinically significant decrease in inflammation [118].

Fibrosis is a pathological characteristic of most chronic inflammatory diseases. Fibrotic tissue is characterized by an abnormal deposition of ECM components such as collagen and fibronectin, which disrupts normal tissue architecture and function [119]. Damage to epithelial or endothelial cells triggered by diverse stressors initially activates the coagulation pathway, followed by acute inflammation and activation of innate immune mediators including resident macrophages, neutrophils, and dendritic cells. Inflammatory and immune mediators cooperate to diminish the cytokines or chemokines and then the inactive fibroblasts were transferred into myofibroblasts [120]. Myofibroblasts may generate additional ECM quantities and exert tractional stresses across the ECM, distorting tissue architecture and eventually leading to fibrosis [120]. hAM could inhibit the abnormal development of fibrotic tissue at various levels, resulting in scarring reduction or absence and maintenance of tissue architecture and functions [121]. A study revealed that hAM extract can transfer differentiated myofibroblasts back into fibroblasts in addition to maintaining the initial fibroblastic phenotype *in vitro* [122]. Moreover, hAM could selectively counteract the effect of TGF-β and promote cell proliferation in keratinocytes by modulating the genetic program [123]. TGF-β can overexpress ECM elements, including metalloprotease inhibitors, collagen, and proteoglycans, which leads to fibrosis in the tissue repair process.

The hAM can induce immunomodulatory properties by inhibiting inflammatory cytokines, decreasing oxidative burst, restricting chemotaxis, and improving phagocytic function [124]. For example, a study found that human limbal epithelial cells cultivated on the ECM of hAM significantly exhibited lower IL-1α/1β mRNA and protein expressions in contrast to plastic cultures, regardless of whether lipopolysaccharide was added or not [125]. In a clinical study, the hAM patches of the ocular surface were applied to 20 eyes of 20 patients with persistent corneal epithelial abnormalities. The results showed that hAM patches attracted and trapped inflammatory cells infiltrating the ocular surface [126]. Therefore, the anti-inflammatory effects of the hAM are speculated to be cytokine-mediated as well as mechanical. In addition, the hAM seems to be immune-privileged tissue containing immunoregulatory factors such as HLA-G and Fas ligand [127]. This low immunogenicity of hAM was also related to low expression of HLA IA, together with negative expressions of immunogenic markers CD40/80/86 and HLA-DR [37]. Consistently, a study conducted *in vitro* demonstrated that CM containing immunoregulatory factors from the intact hAM has immunomodulatory properties in terms of human peripheral blood mononuclear cells and T-cell proliferation, T-cell subset polarization, and T-regulatory cell induction [128].

**Fig. 3:**
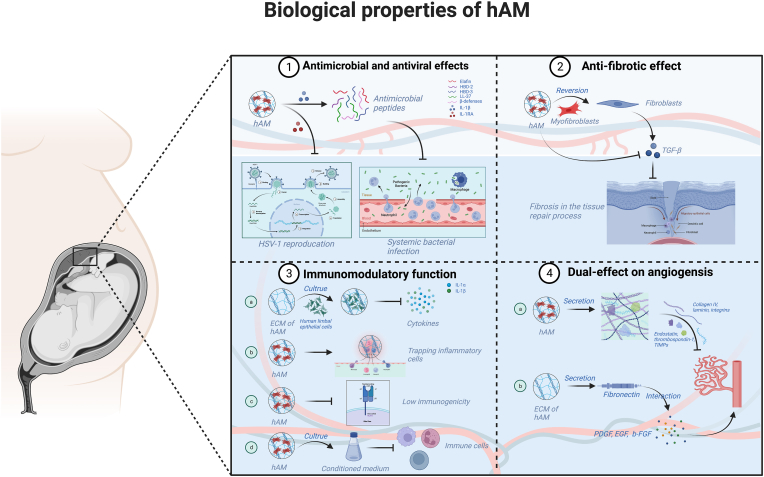
**Schematic representation of distinct features of hAM.** The features include antimicrobial and antiviral effects, anti-fibrotic or anti-scarring activity, immunomodulatory function with low immunogenicity, and pro- or anti-angiogenesis effect.

There are challenging studies about the pro- or anti-angiogenic effects of the hAM. However, a few studies indicated that hAM has a dual-effects on angiogenesis, which is side-dependent [121,129]. In a dorsal skinfold chamber rats model, the number of formed vessels and their lengths increased when hAM was positioned epithelial side, whereas angiogenesis reduced when it was placed stromal side [129]. On the one hand, the hAM contains collagen IV, laminin, and integrins 4/6 that inhibit neovascularization after transplantation, which has been related to angiogenesis suppression in severely damaged rabbit corneas [130]. The hAM also generates other valuable anti-angiogenic proteins, including endostatin, thrombospondin-1, and four tissue inhibitors of metalloproteases (TIMP-1/2/3/4) [131]. When hAM was utilized in post-pterygium operations, it inhibited the blood formation where the fibrous pterygium lesions were located [132]. On the other hand, the fibronectin proteins in the ECM of hAM interact with the growth factors PDGF, EGF, and b-FGF to activate the ERK pathway and then exert its angiogenesis effect *in vitro* [133]. Another study proposed that the hAM might be regarded as an excellent matrix for establishing mature vascular constructions [134]. This is owing to its potential for increasing the expression of platelet-endothelial cell adhesion molecule-1 (PECAM-1) and adhesion molecule VE-cadherin in porcine vascular endothelial cells. In addition, the ECM of hAM that contains hyaluronic acid and proteins, including fibronectin, laminin, collagens, and proteoglycans. This ECM acts as a support cultivation system for cell growth and adhesion [135]. These biological properties of hAM with different forms are diagrammatically displayed in Fig. 3.

## 4 Applications of hAM and its composites in tissue engineering

hAM has been utilized extensively in tissue regeneration and has shown positive outcomes as a reconstruction material. Since the beginning of the 20th century, hAM has shown advantages as a therapeutic agent in clinical applications, primarily in the wound healing of soft tissues like skin and cornea. To date, the hAM has been broadly applied in clinical procedures, including gynecology [136], ophthalmology [137], neurosurgery [138], plastic surgery [139], gastrointestinal surgery [140], and orthopedics [141], as well as wound dressing for burns, or the acute and chronic wounds [121,142]. The clinical applications of hAM also have been reviewed in many publications [[143], [144], [145], [146]]. These articles have investigated unmodified hAM applications. Collectively, hAM, regardless of its state (intact, dried, decellularized, or frozen), has distinct qualities that render it highly suitable for various applications.

However, the fast biodegradability of hAM is considered a severe limitation in its usage for regenerative medicine [15]. Additionally, the biological or mechanical properties of hAM are sometimes not efficient enough for some applications. Therefore, to enhance these characteristics, modifications on hAM with various methods are needed for tissue regeneration. The following section overviews the different modifications based on hAM, both in the intact and decellularized form, as well as their applications in tissue engineering and regenerative medicine.

### 4.1 Eyes

The usage of hAM in treating ophthalmologic diseases has become broadly acknowledged. However, its intrinsic limitations, including weak mechanical characteristics, short-term therapeutic efficiency, difficult suturing during operations, and ineffective adhesion, underline the need for hAM modifications in ocular surface reconstruction [147]. Polyvinyl alcohol (PVA) is a transparent hydrogel that is biocompatible and physically strong, rendering it a viable material for an artificial cornea. Uchino et al. [148] fabricated a hAM-immobilized PVA hydrogel (PVA-AM) to substitute cornea. The results showed that the corneal epithelialization on PVA-AM was improved in rabbit corneas, indicating that PVA-AM could be used as a biocompatible hybrid scaffold for keratoprosthesis. Another study investigated the biodegradability of fibrin glue-coated and freeze-dried hAM (FG-AM) implanted into a rabbit scleral surface without suturing [149]. This FG-AM remained for at least 12 weeks after transplantation. It was verified to be safe, convenient, and effective for ocular surface reconstruction. Cai et al. [147] confirmed that a composite structure of FG-AM transplantation is effective and valuable for ocular reconstruction.

Additionally, Zhou et al. [150] fabricated another composite membrane by electrospinning a layer of poly(-caprolactone) (PCL) nanofiber mesh to strengthen the decellularized hAM sheet by covalent interfacial bonding while preserving the special bioactivity of the decellularized hAM. The authors demonstrated the superior ability of this PCL-AM composite for repairing the damaged cornea in rabbits with limbal stem cell (LSC) deficiency caused by alkaline burn. Notably, biological or chemical components could be coated on the membrane by applying diverse procedures including peptide self-assembly and deposition. These modifications have mainly enhanced the antibacterial property of hAM. For instance, Singh et al. [151] applied a coating of clavanin-A to self-assembled acellular hAM to inhibit biofilm formation on the membrane. The results revealed that the clavanin A-coated hAM was more biocompatible than the decellularized AM alone and exhibited lower levels of fungal and cell colonization. Furthermore, this composite based on hAM had outstanding physical, morphological, and antifungal properties, rendering it appropriate for the controlling ocular surface infections. Our group administered severe corneal injury by AME-derived composites in the form of *in situ* gels or tablets. The results showed that AME could promote wound healing, with the *in situ* gels having the most significant therapeutic efficiency [152].

### 4.2 Skin

Due to the intrinsic bioactive and biocompatible characteristics of hAM, it is believed to have significant therapeutic potential for skin wound healing. However, it is anticipated that hAM reinforcement with a biodegradable polymer would give acceptable mechanical characteristics because it is too soft and thin to handle. Literature [153] reported a fibrin-coated silver nanoparticle-infused poly-[lactide-*co*-glycolide-*co*-caprolactone] terpolymers (PLGC) to strengthen the hAM membrane, together with bioactive chemicals or factors to repair the wound. This hybrid scaffold demonstrated favorable qualities and significant biocompatibility for possible usage in skin wound healing. The ability of the hAM composites as a wound dressing could shield the wound from outside harm and provide an ideal microenvironment for tissue regeneration. Recently, Yang et al. [154] produced a double-layer membrane comprised of decellularized hAM and chitosan sponge as a wound dressing. The results revealed that the porous structure of the composites accelerated blood coagulation and swelling functions, while the bilayer structure exhibited acceptable biocompatibility, particularly for the attachment and proliferation of human foreskin fibroblast cells (HFF-1). After eight-day treatments, the full-thickness skin lesions in the diabetic mice model exhibited more than 80% closure.

Comparable research was conducted to determine whether hAM and collagen-based hydrogels worked to repair cutaneous burn wounds in rats with wound dressing membranes. The results found that when a wound dressing membrane was applied, a hydrogel consisting of hAM and collagen markedly displayed rapid wound healing, driven by full re-epithelialization and closure by wound contraction [155]. Murphy et al. [156] also studied the composites of hAM-based hydrogel, proving the effectiveness of the solubilized hAM combined with the carrier hyaluronic acid hydrogel (HA-SAM) as a wound therapy in a full-thickness wound mice model. The findings showed that HA-SAM dramatically expedited wound closure by re-epithelialization and inhibited wound contraction.

Hypertrophic scarring is a skin disease caused by the deposition of excessive collagen and other ECM proteins after deep trauma, severe burn, and surgical wounds [157,158]. A 3D bilayered decellularized hAM/electrospun silk fibroin membrane was produced and investigated for hypertrophic scarring applications [159]. The results showed a notable reduction in collagen deposition and increased MMP1 expression and deposition in the wound bed in a rabbit ear model, indicating that the manufactured construct can be an effective anti-scarring wound recovery.

### 4.3 Abdominal wall

Laparotomy is a typical surgical technique that may result in complications, such as abdominal infection, adhesions, and hernias [160]. There are 4 million abdominal surgeries are performed annually, and 10–20% of the patients are predicted to develop abdominal hernias at the incision site, which could be regarded as a failure in abdominal repair [161]. Currently, polypropylene (PP) mesh is a synthetic material most frequently utilized to treat incisional hernias and repair abdominal wall defects. However, recurrences and adhesions are frequent complications of abdominal wall reconstruction with a PP mesh [162]. In some cases, hAM is utilized as a coating to improve the therapeutic efficacy of PP mesh. For example, in an abdominal wall defect rat model, PP mesh was covered by fresh hAM to reduce postoperative abdominal adhesions and inflammation while increasing epithelialization after abdominal hernias repair surgery [163]. In another laparotomy rabbit model, the effectiveness of PP mesh coated with hAM could potentially minimize adhesions in the abdominal viscera after laparotomy was performed, compared to PP mesh alone [164]. In addition, Rashid et al. [165] performed a similar study in the abdominal hernias rat model, revealing that the combined bovine amniotic membrane (bAM) and 5% polyethylene glycol (PEG)-4000 effectively prevented the complications generated by PP mesh.

### 4.4 Cardiovascular system

Globally, ischemic heart disease is the leading cause of patient mortality. Myocardial infarction (MI)-related heart failure is linked to severe cardiac remodeling and fibrosis production [166]. A tissue engineering strategy of hAM-based hydrogels has been developed to treat MI. For example, Henry et al. [167] fabricated a thermoresponsive, injectable hAM hydrogel to accelerate myocardial regeneration. They infused hAM-based hydrogel into rat MI hearts via ultrasound-guided injection and examined its effectiveness compared to phosphate-buffered saline. The findings revealed that this hybrid hydrogel markedly increased heart contractility and reduced fibrosis. Another research used an acellular hAM hydrogel modified with polyacrylamide-alginate (AlgSr/PAM-AM) as a vascular biomaterial [168]. The results demonstrated that this vascular graft exhibited the superior mechanical strength, resistance to enzymatic degradation, and anti-calcification properties. Furthermore, it could drastically reduce platelet adhesion, aggregation, activation, and hemolysis. Moreover, this composite hydrogel could substantially stimulate the adherence and growth of endothelial cells as well as vascular tissue regeneration. Similarly, REDV (ArgGlu-Asp-Val) polypeptides were grafted onto double-cross-linked decellularized hAM hydrogel, which demonstrated exceptional mechanical strength, resistance to enzymatic degradation and prevented hemolysis and coagulation [169].

Research is still ongoing for engineered vascular grafts. Aslani et al. [170] successfully produced aligned and random electrospun poly-l-lactic acid (PLLA) scaffolds with hAM lysate surface-coated on them, along with acetylsalicylic acid (ASA) as the anticoagulant. The AM lysate-coated ASA-PLLA-aligned scaffold has shown appropriate tensile strength and proved to support endothelial differentiation, indicating that the scaffolds could significantly develop a biocompatible small-diameter vascular graft.

### 4.5 Orthopedics

Tendon wound is characterized by disordered collagen fibers development, reducing its mechanical characteristics and causing scar production. Instructive scaffolds for tendon regeneration are frequently intended to give mechanical and cellular support. In this case, Hortensius et al. [171,172] fabricated a hAM-based composite structure for tendon regeneration. Collagen-glycosaminoglycan (CG) scaffolds coupled with decellularized hAM was employed to improve tendon wound healing. The scaffolds containing ECM of hAM had dramatically enhanced mechanical qualities, in which the tenocytes had higher metabolic activity even when the medium contained the pro-inflammatory cytokine IL-1β [171]. The fabricated scaffolds impacted the early responses of MSCs to inflammatory stimuli, suggesting that they could be used as a scaffold biomaterial to improve tendon repair [172]. Dewey et al. [173] have developed another composite structure based on decellularized hAM and mineralized collagen scaffold for healing bone wounds. Their results implied that a mineralized collagen-hAM scaffold might be a helpful setting to support craniomaxillofacial bone regeneration, particularly when bone defects displayed severe inflammatory responses.

Besides repairing tendons or bone injury of the composite scaffolds based on hAM, they can also support chondrocyte cultivation. For example, Hussin et al. [174] fabricated a scaffold resembling hyaline cartilage in structure and comprised fibrin and hAM, besides two other biodegradable and biocompatible polymers. The findings demonstrated that this structure had a sufficient biodegradation rate, as well as secreting cartilage-specific ECM and GAGs to preserve cellular phenotype. Furthermore, hAM-based hydrogels may provide sufficient mechanical characteristics for articular cartilage tissue engineering. Toniato et al. [175] have developed a hybrid hydrogel with a high swelling capacity and elastic modulus using hAM, collagen, and chitosan.

### 4.6 Other organs or tissues

**Fig. 4:**
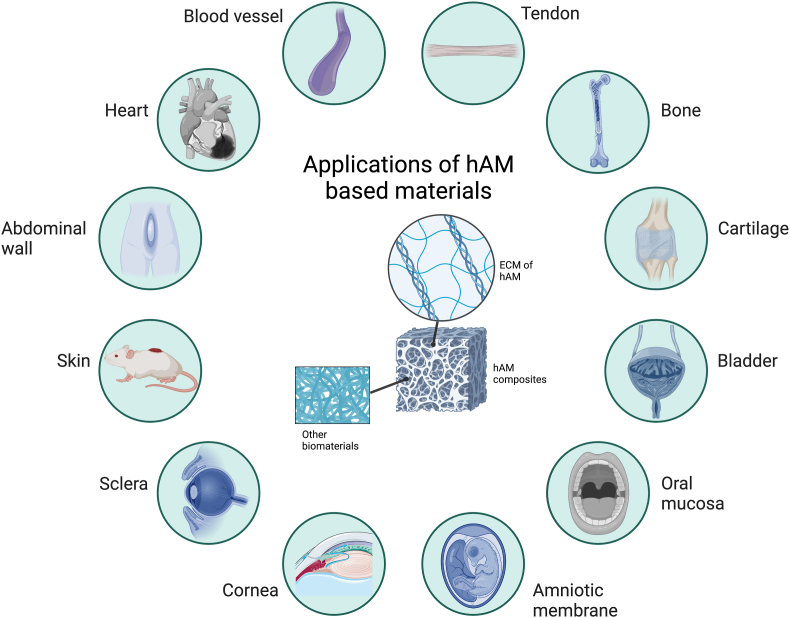
**Therapeutic effects of hAM composites in multiple preclinical studies.** Modifications based on hAM have potential in the eyes, skin, abdominal wall, cardiovascular system, orthopedics, and some other organs.

The applications of hAM in urology reconstruction are limited due to its poor mechanical characteristics. The combination of electrospun nanofibers and hAM provides a novel method for enhancing mechanical resistance while preserving the distinctive bioactivity characteristics of hAM. A sandwich-structured biocomposite material constructed from frozen hAM and the electrospun poly-(l-lactide-*co*-E-caprolactone) (PLCL) was developed to promote bladder wall regeneration [176]. An application of hAM composites for oral mucosal defects is also available. Zhang et al. [177] developed a composite of methacrylate gelatin (GelMA) hydrogel combined with decellularized hAM particles (dAMP) as an oral mucosal substitute to promote mucosa regeneration. The composite substitute (GelMA-dAMP) was easy to synthesize and store for oral mucosal defects repair. The findings showed that neovascularization levels in chick chorioallantoic membranes were significantly promoted, effectively treating oral mucosal defect repair. Another interesting application of hAM composites is the treatment of amniotic membrane defects during pregnancy [178]. The 3D printing technology was used to produce a biocompatible amnion-analogous medical device (AMED) to repair amniotic membrane defects. This construct consists of a polycaprolactone framework and hydrogel generated from decellularized hAM to prevent amniotic fluid leakage and protect fetal survival. In addition, we have conducted a study using decellularized hAM composited with clinical PP mesh to investigate the efficiency of the surgical mesh. The results of *in vivo* tests revealed that the decellularized hAM composited mesh performed excellent biocompatibility, better than the clinical PP mesh in Gynecology surgery [179]. All these applications of hAM-based biomaterials were summarized in Table 4 and vividly exhibited in Fig. 4.

**Table 4:** Applications of hAM-based materials in tissue engineering.

Organs/tissues	Research purpose	hAM state	Composites	Experimental type	Outcomes	Reference
Cornea	Artificial cornea material	Decellularized hAM	PVA-AM	*In vitro* (rabbit corneal epithelial cells)	A biocompatible hybrid scaffold for keratoprosthesis	[148]
Sclera	Ocular surface reconstruction	Freeze-dried hAM	FG-AM	*In vivo* (rabbits)	FG-AM with ideal biodegradability is useful for ocular surface reconstruction	[149]
Cornea	Corneal alkali burns repair	Intact hAM	FG-AM	*In vivo* (rabbits)	FG-AM has more ideal biomechanical properties for ocular surface reconstruction	[147]
Cornea	Corneal alkali burns repair	Decellularized hAM	PCL-AM	*In vivo* (rabbits)	PCL-AM composite can improve re-epithelialization, and reduce inflammation and neovascularization	[150]
Cornea	Corneal infection control	Decellularized hAM	Clavanin A-coated hAM	*In vivo* (rabbits)	Clavanin A-coated hAM with better biocompatibility is appropriate to control ocular surface infection	[151]
Cornea	Corneal injury repair	AME	AME-*in situ* gels or -tablets	*In vivo* (rabbits)	AME with *in situ* gels shows the best treatment efficiency	[152]
Skin	Wound healing	Decellularized hAM	PLGC + silver nanoparticle + fibrin + hAM	*In vitro* (dermal fibroblasts)	hAM, combined with fibrin-silver nanoparticle-PLGC, has excellent biocompatibility for skin regeneration	[153]
Skin	Diabetic wound healing	Decellularized hAM	Double-layer of chitosan sponge + hAM	*In vitro* (HFF-1) and *In vivo* (mice)	Double-layer dressing based on hAM and chitosan has favorable biocompatibility for diabetic wound healing	[154]
Skin	Burn wound healing	Intact hAM	hAM and collagen-based hydrogel	*In vivo* (rats)	Hydrogel based on hAM and collagen accelerates wound healing by covering membranes	[155]
Skin	Wound healing	Solubilized hAM	HA-SAM	*In vivo* (mice)	HA-SAM significantly accelerates wound healing by re-epithelialization	[156]
Skin	Scar minimization	Decellularized hAM	hAM/electrospun silk fibroin	*In vivo* (rabbits)	hAM/electrospun silk fibroin has the potential as an efficient anti-scarring wound dressing	[159]
Abdominal wall	Abdominal wall repair	Fresh hAM	PP mesh covered with hAM	*In vivo* (rats)	PP mesh coated with hAM reduces the abdominal adhesions postoperatively	[163]
Abdominal wall	Abdominal adhesion prevention	Intact hAM	PP mesh covered with hAM	*In vivo* (rabbits)	PP mesh coated with hAM reduces the abdominal adhesions after laparotomy	[164]
Abdominal wall	Abdominal adhesion prevention	bAM	PEG + PP mesh + bAM	*In vivo* (rats)	PP mesh coated with bAM and 5% PEG prevents the abdominal adhesions postoperatively	[165]
Heart	Myocardial infarction repair	Injectable hAM	hAM-based hydrogel	*In vivo* (rats)	hAM-based hydrogel increases heart contractility and reduces fibrosis	[167]
Blood vessel	Vascular graft	Decellularized hAM	AlgSr/PAM-AM	*In vitro* (HUVECs) and *In vivo* (rabbits)	AlgSr/PAM-AM enhances vascular repair	[168]
Blood vessel	Vascular graft	Decellularized hAM	Hydrogel + REDV + hAM	*In vitro* (HUVECs) and *In vivo* (rabbits)	The graft enhances mechanical strength and resistance to enzymic degradation and prevents hemolysis and coagulation	[169]
Blood vessel	Small-diameter vascular graft	hAM lysate	PLLA + ASA + hAM	*In vitro* (HUVECs)	The hybrid scaffold supports endothelial differentiation and provides appropriate biocompatibility	[170]
Tendon	Tendon regeneration	Decellularized hAM	CG + hAM	*In vitro* (tenocytes)	CG combined with hAM enhances mechanical properties and improves tenocytes activity	[171]
Tendon	Tendon injury repair	Decellularized hAM	CG + hAM	*In vitro* (MSCs)	CG combined with hAM improves the inflammatory response of MSCs	[172]
Bone	Bone defect repair	Decellularized hAM	Collagen-hAM	*In vitro* (porcine adipose-derived stem cells)	Collagen-hAM composite scaffold promotes bone repair	[173]
Cartilage	Cartilage tissue engineering	Decellularized hAM	hAM based on fibrin	*In vitro* (chondrocytes)	hAM based on fibrin scaffold can secrete cartilage-specific ECM and GAGs	[174]
Cartilage	Cartilage tissue engineering	Decellularized hAM	Hydrogel (chitosan + collagen + hAM)	*In vitro*	Hybrid hydrogel based on hAM and chitosan, and collagen has a high swelling capacity and elastic modulus	[175]
Bladder	Bladder wall reconstruction	Frozen hAM	PLCL + hAM	*In vivo* (rats)	Frozen hAM sandwiched between two layers of electrospun PLCL promotes bladder wall regeneration	[176]
Oral mucosa	Oral mucosal defect repair	dHAP	GelMA-dAMP	*In vitro* (human fibroblasts) and *In vivo* (rabbits)	GelMA-dAMP is effective for oral mucosal defect repair	[177]
Amniotic membrane	Amniotic membrane defect restoration	Decellularized hAM	AMED	*In vivo* (pig)	AMED prevents amniotic fluid leakage and protects fetal survival	[178]
Uterus	Biocompatibility investigation	Decellularized hAM	PP mesh covered with hAM	*In vivo* (rabbits)	hAM coated with PP mesh shows good biocompatibility	[179]

## 5 Conclusions and perspectives

The research of the hAM has become a prevalent topic for decades. The hAM contains most of tissue engineering components because it can provide the appropriate ECM, stem cells, and other growth factors or cytokines. The unique characteristics of hAM are responsible for its broad applications in clinics in the future. Nowadays, several commercial products are available for clinical applications, especially in ophthalmology and skin wound healing. The hAM-related products will be expanded to various medical fields as high-performance tissue engineering material, benefiting hundreds of millions of patients.

hAM-derived stem cells, including AMSCs and AECs, have significant benefits over other stem cells in abundant sources, easy isolation, minimal ethical issues, no tumorigenicity, and low immunogenicity. Moreover, the biological characteristics of hAM-derived stem cells, especially for the paracrine effects, significantly improved their importance in the experimental study and clinical practice. These characteristics make them a prospective source of stem cells for cell therapy and regenerative medicine. However, more studies are needed to further explore their pluripotent features and the mechanisms of paracrine effects. Further preclinical studies of AMSCs and AECs in treating various disorders should be performed to fully understand their therapeutic prospect.

Although hAM is a fantastic material for regenerative medicine due to its structural, physical, and biological characteristics, it has some drawbacks. These include a high degradation rate, sometimes graft rejection, and relatively poor mechanical characteristics. Therefore, appropriate addressing of these drawbacks can significantly improve hAM applications. Consequently, some modifications are needed to improve its properties combined with other biological, synthetic, or hybrid materials. Limited evidence has confirmed the effectiveness of some modifications in the fields, including ocular surface reconstruction, skin wound repair, abdominal wall reconstruction, and several other applications mentioned in this review. Eventually, more studies are needed to further investigate the effectiveness and importance of hAM-based modified structure.

## Funding

This work was financially supported by 10.13039/501100001809National Natural Science Foundation of China (U20A20121, 32201139) Major Project of 2025 Sci & Tech Innovation of Ningbo (2020Z096), and One Health Interdisciplinary Research Project of 10.13039/501100004387Ningbo University (HY202210), Ningbo.

## Declaration of competing interest

The authors declare that they have no known competing financial interests or personal relationships that could have appeared to influence the work reported in this paper.

## Data Availability

No data was used for the research described in the article.
